# Use of butorphanol as a local anaesthetic for pain management in calves undergoing umbilical hernia repair

**DOI:** 10.3389/fvets.2024.1470957

**Published:** 2024-10-03

**Authors:** Claudia Interlandi, Filippo Spadola, Veronica C. Neve, Marco Tabbì, Simona Di Pietro, Elisabetta Giudice, Daniele Macrì, Giovanna L. Costa

**Affiliations:** ^1^Department of Veterinary Sciences, University of Messina, Messina, Italy; ^2^Experimental Zooprophylactic Institute of Sicily (IZSSi), Palermo, Italy

**Keywords:** calves, umbilical hernia, lidocaine, butorphanol, pain management

## Abstract

The aim of the study was to compare the analgesic efficacy of butorphanol and lidocaine, alone or in combination, in calves undergoing surgical repair of umbilical hernia. The study was conducted in 60 calves of different breeds. Xylazine 0.3 mg/kg was administered intramuscularly to all animals in the study. The animals were then divided into three groups (*n* = 20) that received different treatments with lidocaine at 4.5 mg/kg and butorphanol at 0.02 mg/kg. The L group received lidocaine both by infiltration of the surgical planes and intraperitoneally, the B group received butorphanol both by infiltration of the surgical planes and intraperitoneally, and finally the LB group received lidocaine by infiltration of the surgical planes and butorphanol intraperitoneally. Heart and respiratory rates, haemoglobin oxygen saturation, non-invasive blood pressure and temperature were recorded during surgery. Response to the surgical stimulus was scored on a cumulative numerical scale that included percentage changes in HR, RR and SAP. Postoperative pain was assessed by three independent observers, blinded to treatment, using the UNESP-Botucatu Unidimensional Composite Pain Scale (UNESP-Botucatu UCPS-IV) for the assessment of postoperative pain in cattle. The course of physiological variables was appropriate for patients under anaesthesia. No subject required rescue intraoperative analgesia. In group L, 4 subjects at 40 m and 5 subjects at 50 m required postoperative rescue analgesia. Both butorphanol alone and the combination of butorphanol and lidocaine showed excellent intraoperative and postoperative scores. Furthermore, this combination did not cause any cardiopulmonary or other adverse effects. Based on the results of this study, both butorphanol alone and the co-administration of butorphanol and lidocaine administered locally proved to be safe and effective in providing adequate and long-lasting analgesia in calves, helping to reduce postoperative discomfort and maintaining adequate animal welfare.

## Introduction

A hernia is a protrusion of the contents of a body cavity through an accidental or malfunctioning natural opening. Umbilical hernias are the most common congenital defects in calves with an incidence ranging from 1 to 21% (1). As a result, ventral abdominal surgery for umbilical hernia is one of the most requested procedures in calves (2, 3). The combination of deep sedation and local anaesthesia is the anaesthetic technique of choice for this type of surgery (4). However, in the EU, the list of drugs approved for this purpose in this species is very limited. Although the use of local anaesthetics and opioids administered *in situ* for intraoperative and postoperative pain management is widespread in veterinary medicine, these potent analgesics are still rarely used in cattle.

In damaged peripheral tissues (skin, muscles, joints and viscera), primary afferent neurons (PANs) transduce noxious stimuli into action potentials that are modulated and transmitted to the brain, where they are processed and perceived as “pain” (5). Peripheral opioid receptors (PORs) on PANs represent an important therapeutic target because their inhibition could prevent the transmission of noxious impulses and block the generation of pain in the brain (6, 7).

New multimodal analgesia techniques involve the use of different substances that act synergistically to enhance the effect obtained (8–11).

Butorphanol is an opioid whose mixed agonist–antagonist activity results in analgesia with a lower probability of inducing respiratory depression than pure *μ*-receptor agonist activity, contributing to a balanced anaesthetic management. Butorphanol has been shown to improve superficial and visceral signs of pain in several species when administered intravenously or epidurally (12–16). However, the analgesic and haemodynamic effects of opioids in the bovine species have not been extensively investigated and documented. The aim of the present study was to compare the analgesic efficacy of butorphanol and lidocaine, administered alone or in combination, by infiltration at the surgical incision site and intraperitoneally during umbilical hernia repair in calves. The hypothesis of the study is that locally administered butorphanol, alone or in combination with lidocaine, may improve surgical pain management in this species.

## Materials and methods

### Animals and study design

This study was performed in accordance with Legislative Decree no. 26 of 4 March 2014 on Italian animal welfare legislation and was approved by the Institutional Ethics Committee for Animal Welfare of the University of Messina, protocol number 027/2018. Procedures were performed according to national (Italian Law D.M. 116192) and international (EU Directive 2010/63/EU and USA Public Health Service Policy on Humane Care and Use of Laboratory Animals) regulations for the care and use of laboratory animals. Owners were fully informed and gave written consent for their calves to be enrolled.

Sixty (*n* = 60) calves of different breeds (Friesian, Alpine Brown, Modicana, half-breeds) from different local herds undergoing umbilical surgery were included in this study. The selected calves included 28 males and 32 females and the study was conducted during the spring and autumn seasons, with approximately 6 calves enrolled per month. The inclusion criterion was the presence of an umbilical hernia of 8–13 cm in diameter. The exclusion criterion was the presence of an omphalocele or any other pathological condition. Food was withheld for 7 h prior to surgery and access to water was withheld for 3 h prior to surgery. Calves (*n* = 60) were randomly divided into three groups: Lidocaine, L group (*n* = 20); Butorhanol, B group (*n* = 20) and Lidocaine/Butorhanol, LB group (*n* = 20). Animals underwent umbilical hernia repair surgery at their farms.

### Treatment administration

A prospective, block-assigned, operator-blinded clinical trial was performed on each calf at the farm of origin. On the day of surgery, the animals were weighed (OCS300, Zoo Piro, Cruto, Calabria, Italy) to determine the appropriate dose of drugs. After a 30-min acclimatisation period, xylazine 0.3 mg/kg (Rompun 2%, Bayer, Leverkusen, Germany) was administered intramuscularly (IM) in the box where the operation would be performed. After 15 min, when sedation and muscle relaxation had been achieved, an intravenous catheter (14G × 5″) was inserted to administer lactated Ringer’s solution at a rate of 10 mL/kg/h during surgery. The calves were placed in dorsal recumbency, the umbilical region was aseptically prepared, and a local analgesia protocol was performed. For the local analgesic protocol, the L group received lidocaine 4.5 mg/kg (Lidocaina Cloridrato Esteve 2%, Ecuphar Italia S.r.l., Milan, Lombardy, Italy) both by infiltration of the surgical planes and intraperitoneally, while the B group received butorphanol 0.02 mg/kg (Butorphanol Tartrate, Dolorex 10 mg, Codifa, MSD Animal Health S.r.l., Italy) by infiltration of the surgical planes and intraperitoneally, with the drug dose divided equally between two syringes for both groups. Instead the LB group received lidocaine 4.5 mg/kg only by infiltration of the surgical planes and butorphanol 0.02 mg/kg only intraperitoneally, with each drug administered with its own syringe. To achieve greater diffusion, the volume of each syringe was increased to 40 mL with the addition of saline (0.9% sodium chloride). Infiltration in the umbilical region involved both the skin and muscle planes, while intraperitoneal injection was performed in the hernia sac. Both infiltration of the surgical planes and intraperitoneal administration were performed at different sites.

### Umbilical hernia repair

After sedation, the calves were placed dorsally on a padded mattress and the skin over the umbilical region was cleaned, aseptically prepared and infiltrated. The surgeon performing the surgery was the same for all animals. Open herniorrhaphy (2, 3) was performed through an elliptical skin incision and the adhesions of the parietal peritoneum to the skin were released using both blunt and sharp dissection. After repositioning of the abdominal organs, the hernia ring was refreshed and the horizontal interrupted mattress suture with 2–0 chromic catgut was placed on the peritoneum and supported with an autologous flap from the hernia sac. A single interrupted suture was placed on the subcutis (2–0 chromic catgut) and the skin was conformed to the surgical wound and sutured (2–0 nylon).

### Measurement of physiological parameters

Heart rate (HR), haemoglobin oxygen saturation (SpO_2_, %) and systolic, diastolic and mean arterial pressure (SAP, DAP, MAP) were measured using a multiparameter monitor (EDAN Instruments Italy, Napoli, Campania, Italy). Heart rate and SpO_2_ were measured with a pulse oximeter, while blood pressure was measured with a special cuff placed at the base of the tail, approximately 30–40% of the tail circumference (oscillometric method). Respiratory rate (RR) was determined by counting thoracic excursions per minute. Body temperature was measured using a digital thermometer inserted into the rectum for a few seconds (Digital Veterinary Thermometer, GIMA). Parameters were recorded at T_0_ (basal values) before xylazine administration. Local anaesthesia was then administered at T_1_ (15 min after xylazine administration). Finally, surgery was started at T_2_ (10 min after local anaesthesia administration). From T_2_, parameters were recorded at 5-min intervals: T_3_ (5 min after the start of surgery), T_4_ (10 min), T_5_ (15 min), T_6_ (20 min), T_7_ (25 min), T_8_ (30 min), and T_9_ (35 min) (intraoperative time). Measurements were also taken at T10 upon awakening, when the calf stood. To assess the response to intraoperative noxious stimulation, we used a cumulative numerical scale that considered percentage changes in HR, RR and SAP compared to T_1_ (15 min after xylazine administration) according to the following procedure: (time point value—T_1_ value)/T_1_ value × 100 = % change. Scores were scored as follows: Score 0 = no change; 1 = increase ≤10%; 2 = increase >10% but ≤20%; 3 = increase >20% but ≤30%; 4 = increase >30%. Scores were assigned by assessors blinded to treatment. A final score ranging from a minimum of 0 to a maximum of 12 was obtained by summing the scores of the selected variables. If HR, RR and SAP increased by more than 20%, to a score of 6 or higher, rescue analgesia was administered (11, 17–19), the surgical area was infiltrated and sprayed intraperitoneally with 2 mg/kg lidocaine 2%.

### Post-operative pain assessment

Postoperative pain was assessed by three independent observers, blinded to treatment, using the UNESP-Botucatu Unidimensional Composite Pain Scale (UNESP-Botucatu UCPS-IV) for the assessment of postoperative pain in cattle. Pain scores range from no pain (score 0) to severe pain (score 10). The assessment was made at 10 (T_10_), 20 (T_20_), 30 (T_30_), 40 (T_40_), and 50 (T_50_) minutes after the calves were returned to a standing position. The questionnaire consists of 5 behavioural categories (items) that assess locomotion, spontaneous behaviour, activity, appetite and various behaviours that the animal may exhibit. The items are numbered in ascending order of pain intensity (20–23). The cut-off point for the post-operative pain score was ≥4; if the calf exceeded this value, it received 3.3 mg/kg flunixin meglumine IV (Finadyne, Schering-Plough Animal Health, Oss, The Netherlands) as rescue analgesia.

### Statistical analysis

A sample size calculation was performed to determine the number of cattle required for this study. Sample size was calculated using G*Power 3.1 software (Hein-rich-Heine-Universitat Düsseldorf, Düsseldorf, Germany). An effect size (f) of 0.45, a significance level (*α*) of 0.05, and a power (1-*β*) of 0.85 were assumed using the Anova Fixed Effects, omnibus, one-way test. Statistical analyses were performed using commercially available software (GraphPad Prism version 8.2.1; GraphPad Software Inc., La Jolla, CA, USA, and SPSS version 15.0; SPSS Inc., Chicago, IL, USA). Data were analysed for normality using the Shapiro–Wilk test and reported as mean ± SD or median (range), as appropriate. One-way ANOVA followed by Friedman’s test was used to assess differences between groups for demographic data. Clinical parameters (HR, SpO_2_, SAP, MAP, DAP, RR, T) were analysed by two-way repeated measures ANOVA.

Bonferroni post-hoc pairwise comparison test between least squares means was used when statistical differences were present. Scores were reported as mean +/− standard deviation (SD). To confirm content and construct validity, pain scores related to responses to noxious intraoperative stimulation and the UNESP-Botucatu Unidimensional Composite Pain Scale were summarised as median, minimum and maximum values. Scores were compared within and between groups using Friedman’s test. Kendall’s coefficient of concordance W was calculated to measure the degree of agreement between observers. At each time point measured, the scores of the three observers were averaged. Values of *p* ≤ 0.05 were considered significant for all analyses.

## Results

Sixty-four (*n* = 64) calves with umbilical hernia were studied over a period of 12 months. Of these, four (*n* = 4) were excluded from the study due to co-morbidities. No significant differences in age, weight, body condition score and operative time were found between the calves enrolled (Table 1).

**Table 1:** Demographic data from all groups.

Variable	Group B	Group L	Group LB	*p* value
Weight (kg)	86 (65/99)	88 (75/94)	85 (65/95)	0.55
Age (months)	3 (1/4)	3 (1/4)	3 (1/4)	0.16
Body condition score	4 (3/5)	4 (3/5)	4 (3/5)	0.81
Surgery time (minutes)	46 (40/56)	49 (40/57)	47 (40/56)	0.14

Differences in weight, age, body condition score, and operative time in calves treated topically with butorphanol (B), lidocaine (L) alone, or the butorphanol/lidocaine combination (LB). Results are expressed as median (interquartile range, IQR).

In each group, 20 calves were required to detect a statistically significant a difference, actual power was 0.86. All enrolled animals completed the study, and all recoveries were uneventful. No surgical complications were reported. The interobserver agreement was high (*W* = 1).

Within groups, HR values were statistically lower in the B and LB groups compared to T_0_ at several time points (*p* < 0.001), whereas no significant difference was found in the L group. A statistically significant difference was found between groups for HR values. B group showed a significant decrease compared to L group (*p* < 0.001) and LB group (*p* < 0.001) from T_1_ to T_10_. The LB group had intermediate values between the B and L groups.

Within groups, RR values were significantly higher in the B and LB groups compared to T_0_ at certain time points (T_1_, T_2_, T_9_; *p* < 0.001), whereas a significant decrease was observed in the L group from T_3_ to T_9_ (*p* < 0.001). Among the groups, the B and LB groups showed significantly higher RR values than the L group at certain time points (at T7 with the B group and from T6 to T10 with the LB group; *p* < 0.001). Blood pressure values (SAP, DAP, MAP) during surgery showed a significant reduction compared to baseline (T_0_) at almost all time points in each group (*p* < 0.001). Comparison between groups showed that the B and LB groups had higher blood pressure values than the L group at certain time points (*p* < 0.001). SpO_2_ did not vary significantly between groups at any time point, with optimal values always maintained around 95%. Body temperature did not vary between groups (Table 2).

**Table 2:** Effect of xylazine (0.3 mg/kg IM) followed by: butorphanol (0.02 mg/kg) or lidocaine (4.5 mg/kg) or lidocaine/butorphanol combination administered locally on heart rate (HR), respiratory rate (RR), systolic, diastolic, mean arterial pressure (SAP, DAP, MAP) and body temperature in calves undergoing umbilical hernia repair.

		T_0_	T_1_	T_2_	T_3_	T_4_	T_5_	T_6_	T_7_	T_8_	T_9_	T_10_
HR (beats/min)	B	64 ± 6	52 ± 8^*^	50 ± 6^*^	48 ± 5^*^	49 ± 5^*^	50 ± 4^*^	51 ± 3^*^	53 ± 4^*^	50 ± 4^*^	49 ± 4^*^	52 ± 3^*^
	L	62 ± 6	67 ± 5^♣^	64 ± 6^♣^	67 ± 4^♣^	71 ± 4^♣^	74 ± 2^♣^	75 ± 3^♣^	75 ± 2^♣^	69 ± 6^♣^	69 ± 6^♣^	74 ± 3^♣^
	LB	70 ± 3	61 ± 4^*♣◊^	64 ± 6^*♣^	62 ± 6^*♣^	65 ± 5^♣^	69 ± 4^♣^	70 ± 4^♣^	63 ± 4^*♣^	64 ± 4^*♣^	63 ± 5^*♣^	67 ± 3^♣^
RR (breaths/min)	B	40 ± 5	44 ± 6^*^	48 ± 0.8^*^	41 ± 6	40 ± 7	40 ± 6	39 ± 5	39 ± 4	40 ± 6	37 ± 3	36 ± 4
	L	44 ± 5	48 ± 5	48 ± 7	40 ± 6^*^	41 ± 6^*^	37 ± 4^*^	37 ± 3^*^	33 ± 3^*♣^	36 ± 3^*^	36 ± 4^*^	33 ± 3^*^
	LB	44 ± 5	48 ± 7^*^	50 ± 4^*^	45 ± 5	44 ± 5	41 ± 4	45 ± 4^◊^	45 ± 7^◊^	44 ± 6^◊^	48 ± 6^*♣◊^	45 ± 5^♣◊^
SAP (mmHg)	B	136 ± 9	127 ± 6^*^	112 ± 8^*^	98 ± 7^*^	106 ± 9^*^	103 ± 8^*^	107 ± 10^*^	106 ± 9^*^	110 ± 8^*^	136 ± 9	144 ± 11^*^
	L	136 ± 11	129 ± 11	112 ± 9^*^	102 ± 6^*^	106 ± 7^*^	103 ± 8^*^	102 ± 8^*^	103 ± 9^*^	105 ± 0^*^	110 ± 8^*♣^	104 ± 7^*♣^
	LB	144 ± 10^♣◊^	136 ± 10^*^	114 ± 7^*^	108 ± 5^*♣^	100 ± 5^*^	101 ± 7^*^	99 ± 8^*^	103 ± 8^*^	104 ± 9^*^	112 ± 8^*♣^	115 ± 7^*♣◊^
MAP (mmHg)	B	120 ± 10	98 ± 4^*^	82 ± 7^*^	79 ± 5^*^	81 ± 6^*^	82 ± 6^*^	83 ± 8^*^	88 ± 6^*^	87 ± 5^*^	105 ± 7^*^	124 ± 4
	L	123 ± 4^♣^	117 ± 5^*♣^	83 ± 7^*^	89 ± 3^*♣^	91 ± 3^*♣^	68 ± 9^*♣^	72 ± 8^*♣^	69 ± 8^*♣^	78 ± 6^*♣^	93 ± 3^*♣^	86 ± 4^*♣^
	LB	120 ± 8	106 ± 5^*♣^	91 ± 6^*♣◊^	87 ± 5^*♣^	78 ± 3^*◊^	81 ± 3^*◊^	76 ± 3^*♣◊^	83 ± 3^*♣◊^	82 ± 3^*♣◊^	93 ± 5^*♣^	90 ± 3^*♣◊^
DAP (mmHg)	B	71 ± 3	62 ± 3^*^	59 ± 4^*^	49 ± 4^*^	55 ± 3^*^	50 ± 5^*^	52 ± 3^*^	57 ± 3^*^	58 ± 3^*^	70 ± 4	107 ± 7^*^
	L	76 ± 3	73 ± 3^♣^	55 ± 4^*^	59 ± 4^*♣^	63 ± 3^*♣^	41 ± 3^*♣^	41 ± 2^*♣^	38 ± 1^*♣^	48 ± 3^*♣^	65 ± 5^*♣^	55 ± 4^*♣^
	LB	79 ± 4	71 ± 4^*♣◊^	65 ± 5^*♣◊^	55 ± 9^*♣^	46 ± 5^*◊^	51 ± 3^*◊^	49 ± 2^*♣^	51 ± 3^*◊^	52 ± 3^*^	65 ± 3^*♣^	63 ± 4^*♣^
T° (°C)	B	39.6 ± 1									38.5 ± 5	
	L	39.4 ± 2									38.4 ± 6	
	LB	39.2 ± 1									38.3 ± 4	

^*^Significantly different from baseline within treatment. ^♣^Significantly different from corresponding time point between group B and L or LB groups. ^◊^Significant difference between the L group and the LB group. Two-way ANOVA for repeated measures, followed by Bonferroni test was used to evaluate the changes along the timeline and differences among groups. GraphPad Prism automatically corrects nonparametric data by transforming them into their base 10 logarithms. *p* < 0.05 was considered significant.

The intraoperative noxious stimulation response scale showed a significant reduction from T_3_ to T_10_ compared to baseline in the B and L groups (*p* < 0.001). The LB group showed a significant reduction from T_3_ to T_7_ (*p* < 0.001). Between groups, the B group showed significant differences from the L group at many time points (T_1_, T_2_, T_4_, T_5_, T_6_, T_9_, *p* < 0.001), while the LB group only showed a difference at T_9_ (*p* < 0.01). Comparison between the L and LB groups showed significant differences at T_1_ and T_6_ (*p* < 0.001). In the intraoperative, rescue analgesia was not required in any case (Table 3).

**Table 3:** Cumulative intraoperative score for responses to noxious stimulation.

	T_1_	T_2_	T_3_	T_4_	T_5_	T_6_	T_7_	T_8_	T_9_	T_10_
B	1 (0/3)	2 (0/4)	0 (0/0)^*^	0 (0/0)^*^	0 (0/1)^*^	0 (0/1)^*^	0 (0/1)^*^	0 (0/1)^*^	0 (0/1)^*^	0 (0/1)^*^
L	2.5 (0/4)♣	1 (0/3)^*^♣	1 (0/2)^*^	1 (0/2)^*^♣	1 (0/2)^*^♣	1 (0/3)^*^♣	1 (0/3)^*^	1 (0/3)^*^	1 (0/3)^*^	1 (0/3)^*^♣
LB	1 (0/3)◊	1 (0/4)	0 (0/2)^*^	0 (0/2)^*^	0 (0/1)^*^	1 (0/2)^*^◊	0 (0/2)^*^	0 (0/2)	1 (0/3)♣	0 (0/2)

Values are expressed as median and interquartile ranges (IQR). Percentage change, at various times, in HR, RR and SAP compared to T_1_ (15 min after xylazine administration). ^*^Statistical differences of HR, RR and SAP compared to T_1_ (15 min after xylazine administration) in each group (*p* < 0.001). ^♣^Statistical differences between group B vs. groups L and LB (*p* < 0.001); ^◊^Statistical difference between group L vs. group LB (*p* < 0.001).

**Figure 1:**
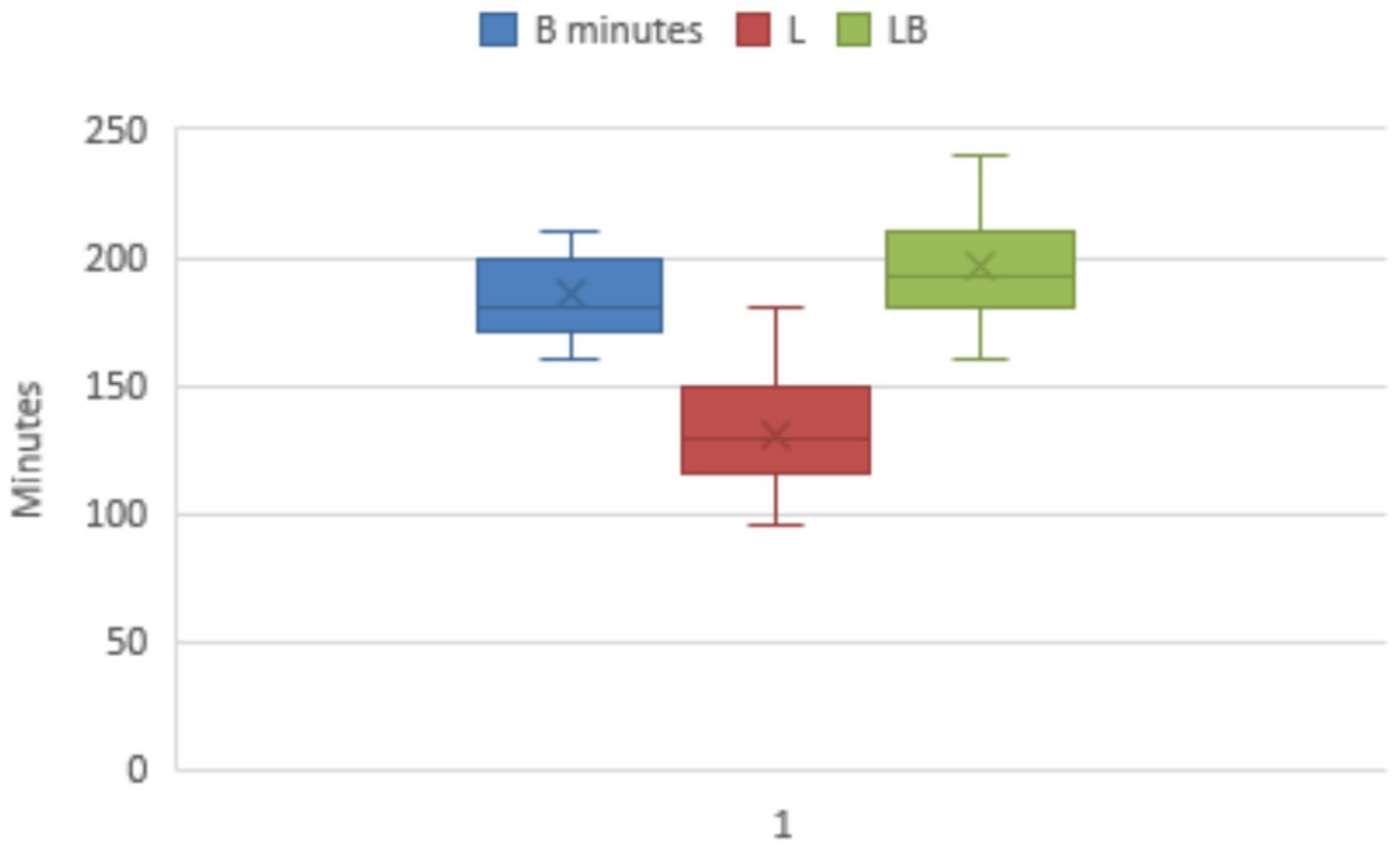
Time from the start of the surgery to the animals’ recovery of the standing position. There was a significant difference between group B and group LB in relation to group L (*p* < 0.000).

The time from start of surgery to recovery of the animals to standing was significantly different between B, LB and L groups (*p* < 0.000) and was 180 min (160/210; 185 ± 15.5) B group, 128 min (95/180; 131 ± 25.6) L group and 192 min (160/240; 196 ± 23.1) LB group (Figure 1).

The assessment of the postoperative pain score using the UNESP-Botucatu Unidimensional Composite Pain Scale showed a significant variation in the B and LB groups at 40 m and 50 m (*p* < 0.001), remaining ≤4 throughout the observation period. In the L group, UNESP-Botucatu showed changes along the time span from 20 to 50 m (*p* < 0.001), with 4 subjects at 40 m and 5 subjects at 50 m requiring rescue analgesia by administration of 3.3 mg/kg intravenous flunixin meglumine (Finadyne, Schering-Plough Animal Health, Oss, The Netherlands). Comparison of the groups with respect to UNESP scores showed a significant difference between B and L (*p* < 0.001) and between LB and L (*p* < 0.001), as the scores of B and LB were lower than those of L throughout the postoperative period (Table 4).

**Table 4:** Results off UNESP-Botucatu Unidimensional Composite Pain Scale for comparision of postoperative pain, performed at 10 (T_10_), 20 (T_20_), 30 (T_30_), 40 (T_40_) and 50 (T_50_) minutes after the calves readopted a standing position.

	Group B	Group L	Group LB		
Minutes	Median (IQR)	Median (IQR)	Median (IQR)	Score min/max	*p*-value
T_10_	0 (0/1)	0 (0/0)	0 (0/0)	(0–10)	n.s.
T_20_	0 (0/1)	1 (0/2)^*♣^	0 (0/0)^◊^	(0–10)	*p* < 0.001
T_30_	0 (0/1)	1 (0/3)^*♣^	0 (0/1)^◊^	(0–10)	*p* < 0.001
T_40_	1 (0/2)^*^	3 (2/4)^*♣^	0 (0/1)^*♣◊^	(0–10)	*p* < 0.001
T_50_	1 (0/2)^*^	3 (3/4)^*♣^	1 (0/2)^*◊^	(0–10)	*p* < 0.001

Scores are reported as median (interquartile range, IQR), minimum and maximum values are reported. ^*^Statistical differences of scores compared to T10 in each group (*p* < 0.001). ^♣^Statistical differences between group B vs. groups L and LB (*p* < 0.001); ^◊^Statistical difference between group L vs. group LB (*p* < 0.001).

## Discussion

Attitudes towards pain management in small animals are evolving and there is ample evidence that pain management is helpful in improving postoperative recovery (11). In contrast, pain management in large animals, particularly cattle, still appears to be suboptimal (24, 25). This is due to several factors, including a lack of knowledge about pain recognition, the belief that cattle have a higher pain threshold than other species, and economic considerations that limit the use of certain drugs (17, 24, 26). In addition, few analgesics are approved for use in food-producing animals (Commission Regulation (EU) no. 37/2010). For these reasons, intraoperative and postoperative pain management in these species is particularly challenging (9). To meet this challenge, extensive research is needed to investigate new practical and cost-effective strategies for pain relief in cattle using analgesic molecules currently available in veterinary medicine (27).

Despite some known adverse effects on the central nervous system (CNS), including sedation, euphoria, dysphoria and arousal, and disadvantages related to the cost and regulation of their possession, opioids are the most effective analgesics available for pain management in veterinary medicine (28). New insights in recent years into the peripheral endogenous opioid system (PEOS) offer the possibility of developing new therapeutic strategies to exploit the analgesic effect of opioids, while minimising adverse systemic effects. The PEOS consists of peripheral opioid receptors (PORs) and peripheral leukocyte-derived opioids (PLDO). Tissue lesions and associated inflammation, such as during surgical tissue dissection, increase the concentration of PLDO-secreting leukocytes, but also the number and efficacy of PORs expressed on primary afferent neurons (PANs) (28). This upregulation of PORs is accompanied by sprouting of new peripheral sensory nerve terminals, alteration of the perineural barrier and reduction of pH. Taken together, these mechanisms enhance the interaction between opioid receptors and G-proteins, thereby increasing the antinociceptive efficacy of opioids in peripheral tissues (29–31). Several experimental and clinical studies have demonstrated the peripheral efficacy of opioids. For example, preservative-free morphine can be administered to canine and equine joints after arthroscopy or arthrotomy to provide analgesia via PORs (28). Other studies have shown that local application of the opioid receptor agonists *μ* (MOR), *δ* (DOR) and *κ* (KOR) produces significantly greater analgesia in injured tissue than in healthy tissue, both in animal models and in humans (32–34). Furthermore, while endogenous analgesia is mediated by both central and peripheral opioid receptors in the early hours, it is predominantly mediated by PORs in the later phases (35). Thus, the analgesic efficacy of peripheral opioids increases significantly with the duration of tissue injury, as observed in animal models of neuropathic, visceral, thermal, bone and oncological pain (6).

Although not a traditional local anaesthetic, in this study we wanted to investigate the potential local analgesic efficacy of butorphanol. This is the first study to investigate the use of locally butorphanol alone or in combination with lidocaine in calves sedated with xylazine for umbilical hernia surgery. The results of the present study suggest that both butorphanol and the butorphanol-lidocaine combination may provide satisfactory intraoperative and postoperative pain management and may therefore be a reasonable alternative to lidocaine alone for maintaining analgesia in calves undergoing routine surgery such as umbilical hernia repair.

Opioids are commonly used in multimodal analgesic regimens in veterinary medicine to improve pain relief, and combination with an alpha-2 agonist enhances the effect (36). Butorphanol is an opioid that produces analgesia through its *κ*-receptor partial agonist and *μ*-receptor antagonist actions, which are particularly important for pain management in calves (27). The pharmacokinetic and pharmacodynamic, cardiovascular effects and analgesic activity of butorphanol in calves are poorly reported since most studies have evaluated its adjuvant effects in combination with sedatives (alpha-2 agonists such as xylazine and detomidine), analgesic-dissociative drugs (ketamine) and inhalational anaesthetics (sevoflurane and isoflurane) (8–10, 27, 37).

The pharmacological properties and analgesic efficacy of butorphanol when administered alone have only recently been studied (9). Quantitative evaluation of antinociceptive activity in healthy calves confirmed a statistically significant antinociceptive effect of butorphanol, associated with marked arousal. Co-administration of detomidine abolished the excitatory effect and induced significant sedation, enhancing the antinociceptive effect of butorphanol and the resulting analgesia. However, the authors hypothesised that the mild antinociceptive effect of butorphanol alone, when administered systemically, would not be sufficient during surgical procedures performed routinely in cattle (9).

For this reason, although it remains unclear whether butorphanol alone can affect heart rate, its combination with sedatives is necessary to achieve adequate levels of analgesia and requires constant and careful monitoring of cardiorespiratory parameters. The combination of butorphanol with xylazine reduced the doses required for effective analgesia and increased the overall sedative effect (9, 10). In our study we did not observe any excitatory behaviour in calves treated with topical butorphanol. The time from the start of surgery to recovery of the upright position was different in the three groups, with groups B and LB recovering the upright position in a longer time than group L, which recovered the upright position in a shorter time, which could be related to a potentiating effect of butorphanol with the alpha2-agonist (10, 38).

When xylazine was co-administered with lidocaine in a distal paravertebral block, a significantly longer duration of anaesthesia was observed compared with lidocaine alone. Our results are consistent with previous studies showing that the addition of an alpha2-adrenoceptor agonist also prolongs the duration of local anaesthesia after epidural administration in various species (39, 40). It is likely that the lower scores and longer recovery time of the quadrupeds in groups B and BL were due to the systemic absorption of butorphanol after local injection. Previously, some authors reported that the use of butorphanol (0.1 mg/kg) in combination with IM xylazine (0.2 mg/kg) provided good pain control in calves between 4 and 6 weeks of age (41). In contrast, other authors reported that calves sedated with IM xylazine (0.7 mg/kg) and blocked with procaine showed clear signs of pain (42).

Intravenous administration of butorphanol at 0.2 mg/kg to calves anaesthetised with 3.7% sevoflurane was associated with a decrease in heart rate (HR) and blood pressure (SAP, MAP and DAP) (36), whereas administration of CRI (constant rate infusion) at 20 μg/kg/min to calves anaesthetised with 1.4% isoflurane did not produce clinically relevant changes in haemodynamic values (8). Several authors have reported a statistically significant decrease in heart rate after intravenous administration of various alpha-2 agonists and opioids (9, 10, 22); this effect was also observed in our study with the use of xylazine. The mean HRs for treatment B were significantly lower during the observation period than for the other treatments (groups L and LB). The observed change was relatively small, and bradycardia was not observed in any subject in group B. The normal range of bovine heart rate is reported to be between 38 and 96 bpm, and although the subjects monitored were calves, they were within these parameters (39). Therefore, this result may be insignificant from a physiological and clinical point of view. A significant increase in RR has been described when butorphanol is injected into the subarachnoid space (43) and our results also showed an increase in RR at certain time points in the B and LB groups.

One study evaluated the efficacy of combining morphine with lidocaine and ketamine in calves undergoing routine umbilical herniorrhaphy, with good results in patient management and adequate postoperative analgesia, but the cost and technical support to monitor and maintain CRI (constant rate infusion) makes this protocol infeasible in the field (44). Adverse behavioural effects of butorphanol have been observed in horses, including ataxia and stimulation of locomotor activity. The effects are transient and dose-dependent and are mainly observed after intravenous bolus injections of high doses (0.1 to 0.5 mg/kg); in fact, the same effects were minimised during continuous infusion compared with a single butorphanol injection (16). Other studies have observed that intravenous butorphanol (0.1 mg/kg) has analgesic potential in neonatal and older foals, with no apparent adverse behavioural effects, for the management of painful somatic conditions (45). In our study, we did not observe any side effects in calves, which may be related to the mode of administration of the drug and its wide tissue distribution, typical of opiates, which mainly determines its effect at the site of administration. Determining the cut-off point for rescue analgesia is an additional requirement to assist the veterinarian in making appropriate clinical decisions regarding analgesic therapy in the postoperative period (22). Recognition and measurement of postoperative pain are therefore critical in determining the need for and effectiveness of postoperative analgesia and rescue analgesia. Several scales for the assessment of pain in farm animals such as cattle, sheep and pigs have been reported in the literature. Among the different scales, the UCAPS (UNESP-Botucatu Unidimensional Composite Pain Scale for assessing postoperative pain in cattle), the USAPS (UNESP-Botucatu Sheep Acute Composite Pain Scale) and the UPAPS (UNESP-Botucatu Pig Composite Acute Pain Scale) showed the highest overall strength of evidence for construct validity, criterion validity and reliability (46). The UNESP-Botucatu unidimensional scale for the assessment of postoperative pain is a valid, reliable and repeatable instrument that has been used in both cattle (20, 23) and other species such as pig (47), horse (48) and cat (49). In this study, a cut-off score of ≥4 on the Botucatu Unidimensional Composite Pain Scale was chosen *a priori* to resort to post-operative rescue analgesia. This score was established considering the clinical assessment, even if the score was below the established cut-off point (20, 23). Only in a few subjects treated with lidocaine (group L) did we have to resort to rescue anaesthesia at T_40_ and T_50_; this may be related to the reduction/disappearance of the effect of the local anaesthetic.

It has been reported in the literature that lower ambient temperatures lead to a greater decrease in body temperature in subjects after sedation (50). Subjects in all groups experienced a decrease in temperature, but the parameters remained within optimal ranges, probably because our study was conducted in mild environmental conditions (average daily ambient temperature of around 18°C) (51, 52). Thus, in our case, sedation with xylazine seems to be appropriate for calves to avoid the detrimental effects of cold stress and could help prevent calves from contracting diseases such as respiratory infections or diarrhoea shortly after surgery (38, 53). Cagnardi et al. compared the sedative effects and pharmacokinetics of intravenous dexmedetomidine with those of xylazine. The results obtained were comparable to those observed with xylazine. We can therefore hypothesise that the use of other alpha-2 agonists may also be associated with the local administration of butorphanol (54).

Despite the encouraging results, this preliminary study has some limitations. The variable amount of hernial adhesions observed among the animals and the resulting variability in the surgical manipulations required may have influenced the amount of noxious stimuli the animals were exposed to. The lack of a control group with intramuscular butorphanol prevents direct comparison with local administration in terms of analgesic efficacy and adverse effects. The lack of monitoring of plasma levels of butorphanol after local administration prevents verification of possible systemic absorption and assessment of the elimination period. Regarding the management costs of the protocol presented in this study, the authors do not believe that the use of butorphanol alone or in combination with lidocaine will increase therapeutic costs, given the savings in analgesic or anti-inflammatory drugs in the postoperative period and the low doses used, which have been shown to be effective in pain management.

## Conclusion

The results of this study suggest that local administration of both butorphanol alone and the butorphanol-lidocaine combination may be a viable alternative for intraoperative and postoperative pain management, and thus maintaining an adequate level of comfort, in calves undergoing surgery. Both butorphanol alone and the butorphanol-lidocaine combination at the doses used in this study produced effective analgesia in terms of intensity and duration, as evidenced by optimal intraoperative and postoperative scores. In addition, both treatments were safe, with no cardiopulmonary, excitatory or other adverse effects. Further research is needed to fully understand the pharmacodynamics and pharmacokinetics of butorphanol when administered locally, to establish a dosage range, and to determine potential applications in other types of surgery or other production categories in this species.

## Data Availability

The raw data supporting the conclusions of this article will be made available by the authors, without undue reservation.
